# Health related quality of life in untreated and treated patients with AIS. Study I: back pain, curve magnitude and trunk appearance

**DOI:** 10.1186/1748-7161-9-S1-O81

**Published:** 2014-12-04

**Authors:** Manuel Rigo, Monica Villagrasa, Elisabetta DAgata

**Affiliations:** 1 Elena Salva Institut, Barcelona, Spain; 2 Institut Reçerca Vall Hebron, Barcelona, Spain

## Background

Pain or fears about future pain is one of the main reasons for medical consultation. Patients and families use to believe that Cobb angle and back pain are linearly related. The relationship between scoliosis magnitude, HRQL and Self Perception of Trunk Deformity is not clear. To better understand this, a prospective data collection including age, main thoracic and lumbar or thoracolumbar Cob angle, Trunk Asymmetry Perception Scale TAPS, SRS-22 and current treatment was started involving all patients with idiopathic scoliosis attending a rehabilitation clinic, 10 years of age or older at the time of consultation. The data have been retrospectively analyzed and are presented in several studies. This is the study I.

## Purpose

The aim of this study was to analyze the relationship between back pain (SRS-22 Pain), curve magnitude and TAPS.

## Methods

N=240; mean age 19.3 y + 10.3 (10-62), mean Cobb thoracic 33.7º + 13.8, lumbar or thoracolumbar 29.7 + 12.7. At the time of consultation patients were untreated or treated (exercises, RSC brace + Scoliosis Physiotherapy Exercises, other braces, surgery). The sub-population of untreated patients was also analyzed, N=76, mean age 19.6 y + 11.6, mean Cobb thoracic 28.2º + 14.8, lumbar 25.2º + 14.7. SPSS was used for statistics.

## Results

In the whole sample there was no correlation between SRS-22 Pain and the Cobb angle of the main thoracic curve; a significant but weak correlation was found between SRS-22 Pain and Cobb Lumbar (r= -.22 p< .005), TAPS (r= .36 p<.001) and age (r=-.49 p<.001). In the sub-population of non-treated patients, SRS-22 Pain correlated with Cobb Thoracic (r= -.35 p= .01), TAPS (r= .43 p<001) and age (r= -.62 p>.001), but not with Cobb lumbar.

## Discussion and conclusions

Back pain is multi-factorial. Age and subjective perception of trunk deformity show both a stronger linear relationship with back pain than the Cobb angle. The weak linear relationship existing between back pain and thoracic Cobb angle in untreated patients disappear in the whole sample (untreated and treated patients) and the opposite for the Lumbar Cobb. Interpretation is complex trough out study I.

